# Highly Dispersed N-Doped Graphene Quantum Dot-Assisted NiFe Bimetallic Sites for Efficient Water Oxidation

**DOI:** 10.3390/ma19102081

**Published:** 2026-05-15

**Authors:** Yongbo Wang, Xin Jin, Yanfei Fan, Guanwei Cui, Bo Tang

**Affiliations:** 1 College of Chemistry, Chemical Engineering and Materials Science, Shandong Normal University, Jinan 250014, China; wyongbo1011@163.com (Y.W.);; 2 Laoshan Laboratory, Qingdao 266100, China

**Keywords:** N-GQDs, NiFe-LDH, non-noble metal catalysts, OER, electrocatalysis, green hydrogen

## Abstract

Electrochemical water splitting for hydrogen production is a key technological route toward the large-scale generation of green hydrogen. However, the anodic oxygen evolution reaction (OER) suffers from sluggish kinetics and high overpotential, necessitating the development of non-noble metal catalysts that simultaneously possess low cost, high activity, and excellent stability. In this work, a nitrogen-doped graphene quantum dots@nickel–iron layered double hydroxide (N-GQDs@NiFe-LDH) composite catalyst was in situ constructed via a facile hydrothermal strategy. Benefiting from the electronic modulation and structural confinement effects of N-GQDs, the intrinsic catalytic activity and structural stability of the catalyst were simultaneously enhanced. The as-prepared catalyst requires an overpotential of only 320 mV to deliver a current density of 500 mA cm^−2^ and maintains 98% of its initial activity after 100 h of chronoamperometric stability testing, demonstrating promising potential for practical applications. Multiscale characterizations revealed that N-GQDs formed strong electronic interactions with Ni/Fe active sites at the interface, significantly enhanced interfacial electron transport, and accelerated the OER kinetics. This study demonstrates that the N-GQDs@NiFe-LDH catalytic system constructed via an interfacial heterostructure engineering strategy provides a new insight for the rational design and development of efficient non-noble-metal OER electrocatalysts.

## 1. Introduction

Hydrogen energy, as an ideal emerging clean energy, possesses prominent advantages including high combustion calorific value, extensive sources and zero carbon emission [1,2]. It is recognized as the core energy to underpin future low-carbon energy frameworks and accomplish global carbon neutrality goals [3,4]. Compared with traditional coal gasification hydrogen production, electrochemical water splitting for hydrogen production features a simpler process and yields ultra-high-purity hydrogen, representing the mainstream large-scale technical pathway for green hydrogen production [5,6]. The electrochemical water splitting reaction is composed of two coupled half-reactions: cathodic hydrogen evolution reaction (HER) and anodic oxygen evolution reaction (OER). Among them, anodic OER undergoes complicated four-electron transfer processes, accompanied by sluggish intrinsic reaction kinetics and high reaction energy barriers. Consequently, OER dominates the rate-determining step of overall water splitting, which remarkably suppresses the energy efficiency of water electrolysis [7]. Currently, commercial OER electrocatalysts are predominantly RuO_2_- and IrO_2_-based materials. Although these materials exhibit excellent catalytic activity, precious metals suffer from scarce natural reserves, high economic cost and unsatisfactory long-term operational stability (e.g., dissolution of active components, surface reconstruction), making large-scale practical application extremely difficult [8]. Therefore, it is imperative to develop low-cost non-noble OER electrocatalysts with superior intrinsic activity and structural stability, which can promote overall efficiency of electrolytic water hydrogen production possesses vital scientific research significance and application value for the industrial popularization of green hydrogen.

Transition metal compounds (TMCs) have been extensively investigated as ideal electrocatalytic materials by virtue of their abundant earth reserves, low cost and controllable structural properties [9,10]. Among them, nickel–iron layered double hydroxides (NiFe-LDHs) possess typical two-dimensional layered nanostructures, high electrical conductivity, sufficient active sites and optimal electronic structure, making them the most widely studied promising anodic alternatives to noble-metal catalysts [11,12]. Precise regulation of interfacial electron configuration, optimization of surface coordination environment and interfacial interactions can efficiently decrease reaction energy barriers and accelerate charge transfer rate, thereby enhancing the intrinsic activity and stability of NiFe-LDH-based catalysts. Common modification strategies currently include elemental doping [13,14], lattice defect engineering [15], heterojunction interface construction [16] and lattice strain modulation [17]. And these methods can regulate the coordination environment of active sites and interfacial electron transport behavior, thus facilitating the kinetic process of OER catalysis. Nevertheless, NiFe-LDH tends to undergo irreversible lattice reconstruction, dissolution of active metal ions and collapse of layered frameworks during long-term high-current operation, giving rise to obvious attenuation in catalytic performance [18]. Therefore, developing NiFe-LDH catalysts with high intrinsic catalytic activity and excellent stability possess great scientific significance and practical application value, whereas severe challenges still remain to be addressed.

Graphene quantum dots (GQDs), typical zero-dimensional graphene fragments, consist of sp^2^/sp^3^ hybridized carbon cores and amorphous shells abundant in functional groups (e.g., hydroxyl, carboxyl, amino, pyrrole nitrogen, pyridine nitrogen) [19]. The surface chemical properties and electronic structures of GQDs are collectively dominated by particle size, defect configuration, heteroatom doping modes and surface functional groups [19]. Numerous studies have demonstrated that N-GQDs exhibit enormous application potential in electrocatalysis owing to their high electrical conductivity, distinctive quantum confinement effect, abundant surface functional groups and defect active sites [20]. N-GQDs can serve as nano-supports and electronic modulation units, which afford highly dispersed metal anchoring sites, suppress aggregation of metal active centers, strengthen interfacial electronic interactions and accelerate interfacial electron transfer kinetics [21]. Xia et al. [22] reported a universal synthesis strategy for high-loading transition-metal, single-atom catalysts by employing amino-functionalized GQDs as substrates, realizing high-density immobilization of Ir, Pt, Ni and other single atoms, along with enhanced catalytic activity and durability toward the CO_2_ reduction reaction. In addition, N-GQDs act as interfacial modifiers. Their abundant surface functional groups effectively reduce interfacial impedance, shorten diffusion paths of electrolyte ions and improve mass transport dynamics during catalytic reactions. Song et al. [23] fabricated bifunctional electrocatalysts of amorphous cobalt phosphide modified by halogen-doped carbon dots via a hydrothermal method. Among them, urchin-like F-CDs/CoP/NF displayed exceptional HER/OER performance and long-term operational stability in alkaline electrolytes. However, investigations regarding the application of N-GQDs in NiFe-LDH catalytic systems remain relatively scarce, and systematic explorations into N-GQDs’ regulated electronic structure, interfacial coupling mechanism and catalytic reaction kinetics are still insufficient.

Herein, we rationally designed and in situ constructed a N-GQDs@NiFe-LDH, denoted as N-F1N2 electrocatalyst, via a facile two-step hydrothermal method. In this system, N-GQDs serve as dual-functional units for electronic modulation and structural confinement, enabling optimization of the local electronic configuration of Ni/Fe bimetallic sites and stabilization of the layered structure of NiFe-LDH. The results reveal that the introduction of N-GQDs is expected to promote the formation of high-valence active species, enhance interfacial charge transfer, and effectively anchor the layered lattice of NiFe-LDH to suppress irreversible reconstruction and metal ion leaching under large-current conditions. This work not only aims to develop a high-performance, non-noble-metal OER electrocatalyst with superior activity and exceptional stability for electrochemical water splitting but also proposes a universal interfacial engineering strategy for NiFe-LDH-based catalysts, offering new insights into the rational design of advanced electrocatalysts for large-scale green hydrogen production.

## 2. Experimental Section

### 2.1. Materials

Citric acid (CA), ethylenediamine (EDA), Ni(NO_3_)_2_·6H_2_O, Fe(NO_3_)_3_·9H_2_O, urea (CH_4_N_2_O), ammonium fluoride (NH_4_F), KOH and absolute ethanol were obtained from Sinopharm Chemical Reagent Co., Ltd. (Shanghai, China). Acetone and concentrated hydrochloric acid (HCl) were purchased from Yantai Yuandong Fine Chemical Co., Ltd. Nickel foam (NF, pore size: 110 ppi, thickness: 2 mm, Laiyang, China) was supplied by Kunshan Guangjiayuan New Materials Co., Ltd. (Suzhou, China) Ultrapure water (18.2 MΩ·cm^−1^) was prepared by a laboratory ultrapure water machine.

### 2.2. Preparation of N-GQDs

A hydrothermal bottom-up strategy was employed to synthesize N-GQDs, and the detailed experimental processes were consistent with our previous reported work [24].

### 2.3. The Preparation of N-F1N2 Electrocatalysts

**Figure 1:**
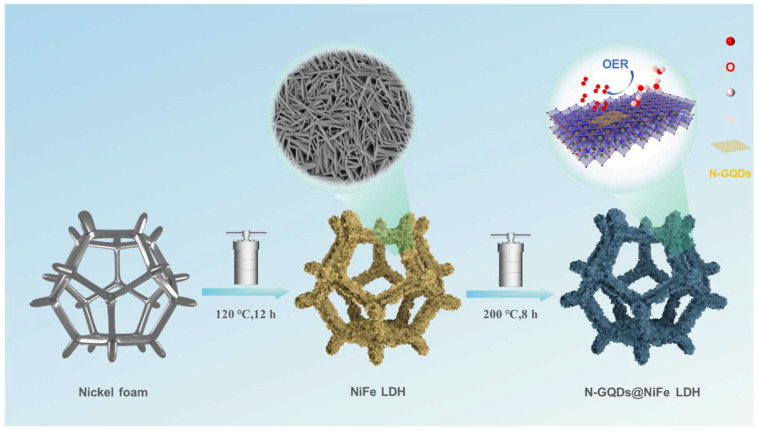
Schematic illustration of the synthesis procedure of N-F1N2.

NiFe-LDH was in situ grown on NF substrates by a facile hydrothermal method, as shown in Figure 1. Firstly, NF was sequentially ultrasonicated in 1 M HCl, ethanol and deionized water for 15 min to remove surface oil contaminants and nickel oxide. Subsequently, 193.9 mg Ni(NO_3_)_2_·6H_2_O, 134.7 mg Fe(NO_3_)_3_·9H_2_O, 300 mg CH_4_N_2_O and 148.2 mg NH_4_F were dissolved in 30 mL deionized water. The homogeneous solution was transferred into a 50 mL Teflon-lined autoclave, and the pretreated NF was subsequently immersed in the solution. The hydrothermal reaction was conducted at 120 °C for 12 h. After cooling to room temperature, the samples were washed thoroughly with ethanol and deionized water, followed by vacuum drying at 40 °C for 12 h. The resulting product was denoted as F1N2. To investigate the effect of Ni/Fe molar ratios on electrocatalytic performance, the total amount of metal nitrates was fixed at 1 mmol. Samples with different Ni/Fe molar ratios (0:1, 1:1, 1:2 and 1:0) were prepared and labeled as Fe, F1N1, F2N1 and Ni, respectively. The mass loading of the catalysts was estimated to be approximately 3.5 mg cm^−2^, based on repeated measurements using the weight difference method.

For the synthesis of N-GQDs@NiFe-LDH, a 15 mL N-GQD dispersion with a concentration of 0.25 mg mL^−1^ was ultrasonically dispersed for 30 min. Subsequently, F1N2 was treated with the N-GQD solution in a 25 mL autoclave at 200 °C for 8 h. The products were washed several times with deionized water and absolute ethanol and then vacuum-dried at 40 °C for 12 h. The final composite catalyst was designated as N-F1N2. And the mass loading of N-F1N2 was estimated to be approximately 3.5 mg cm^−2^, indicating that the secondary hydrothermal treatment did not induce an obvious change in catalyst loading.

### 2.4. Material Characterizations

The micromorphology of materials was characterized by field emission scanning electron microscope (FESEM, SU8010, Hitachi, Ltd., Tokyo, Japan). Prior to characterization, the samples were sputter-coated with gold for 15 s to improve conductivity. Elemental distribution on sample surfaces was analyzed via energy-dispersive X-ray spectroscopy (EDS, Ultim Max 100, Oxford Instruments, Concord, MA, USA). Transmission electron microscopy (TEM) was performed on a JEM-F200 microscope (JEOL Ltd., Tokyo, Japan) employed to investigate the crystal structure at an accelerating voltage of 200 kV. Selected area electron diffraction (SAED) patterns and lattice fringes were analyzed and calibrated using Digital Micrograph 3 software (version 3.53.4141.0, Gatan Inc., Pleasanton, CA, USA). X-ray diffraction (XRD) patterns were collected using a Smart Lab SE diffractometer (Nippon Rikyu Denki Instrument Co., Ltd., Tokyo, Japan) with Cu Kα radiation at a scanning rate of 5° min^−1^ over the 2θ range of 10–80°. Raman spectra were recorded on a LabRAM HR Evolution spectrometer (HORIBA, Kyoto, Japan) using a 633 nm laser in the wavenumber range of 100–2000 cm^−1^ to investigate the surface structure. X-ray photoelectron spectroscopy (XPS) measurements were performed on an ESCALAB 250 instrument (Thermo Fisher Scientific, Waltham, MA, USA) to analyze the surface elemental valence states and elemental composition. All XPS spectra were calibrated using the C 1s peak at 284.8 eV as the reference. Inductively coupled plasma optical emission spectrometry (ICP-OES, iCAD7400, Thermo Fisher Scientific Co., Ltd., Waltham, MA, USA) was used to quantitatively analyze the leached metal ions in the electrolyte, and nitric acid was adopted to adjust the electrolyte to neutral or weakly acidic conditions.

### 2.5. Electrochemical Measurements

Electrocatalytic measurements were conducted in a standard three-electrode system using 1 M KOH as the electrolyte on a CHI660 electrochemical workstation (Shanghai Chenhua Instrument Co., Ltd., Shanghai, China). Catalyst-loaded NF (working area: 1 × 1 cm^2^) served as the working electrode, with a carbon rod and Hg/HgO electrode as the counter and reference electrodes, respectively. All potentials were converted to the reversible hydrogen electrode (RHE) according to the Nernst Equation (1):

E(RHE) = E(Hg/HgO) + 0.098 + 0.059 × pH (1)

Prior to electrochemical measurements, the working electrodes were activated by cyclic voltammetry (CV) for 30 cycles at a scan rate of 50 mV s^−1^ within the potential window of 0–0.8 V. Linear sweep voltammetry (LSV) curves were recorded at 5 mV s^−1^. All polarization curves were subjected to 90% iR compensation. Electrochemical impedance spectroscopy (EIS) measurements were performed at open-circuit potential over frequencies ranging from 10^−1^ to 10^5^ Hz with an amplitude of 5 mV.

CV curves at various scan rates (40–120 mV s^−1^) were measured in the non-Faradaic potential region (0.9–1.2 V vs. RHE). The double-layer capacitance (Cdl) was calculated from the linear relationship between current density and scan rate, and the electrochemical active surface area (ECSA) was subsequently estimated using Equation (2) [25]:

ECSA = Cs/Cdl (2)

Herein, the specific capacitance Cs of alkaline metal hydroxides was set as 0.04 mF cm^−2^, and Cdl corresponds to the fitting slope of current density versus scan rate.

Chronopotentiometry was adopted to evaluate catalytic durability. The catalyst was continuously operated for 100 h at 500 mA cm^−2^, and the time-dependent current variation was recorded to evaluate long-term operational stability under large-current conditions. Chronoamperometric stability tests were performed at a constant potential without iR compensation unless otherwise specified.

## 3. Results

### 3.1. Structural Evolution and Formation Mechanism of N-F1N2 Composites

Figure 1 illustrates the synthetic schematic diagram of N-F1N2. N-GQDs were fabricated via hydrothermal reaction, filtration, dialysis and freeze-drying using CA as the carbon source and EDA as the nitrogen source, and the corresponding reaction mechanism has been described in our previous research [26]. Pristine NF possesses dense passivating NiO layers and residual oil contaminants on the surface, which hinder the in situ growth of NiFe-LDH. The abundant defects are generated on the NF surface after ultrasonic acid etching with 1 M HCl, which serve as nucleation sites for the in situ growth of NiFe-LDH nanosheets, facilitating the uniform growth of layered nanosheets on NF substrates. During the hydrothermal process, urea serves as an alkaline source to slowly release OH^−^ ions. Homogeneous coprecipitation reactions occur between OH^−^ and Ni^2+^/Fe^3+^ ions, forming two-dimensional Ni-Fe hydroxide octahedral laminar structures via coordination interaction. Fe^3+^ ions uniformly substitute Ni^2+^ in the laminar lattice via isomorphous substitution, endowing metal hydroxide layers with excess positive charges. Meanwhile, CO_3_^2−^ anions derived from urea hydrolysis act as interlayer balancing anions to neutralize interlayer charges through electrostatic interactions. Consequently, ordered stacking and oriented growth of two-dimensional laminates produce well-crystallized and structurally stable NiFe-LDH nanosheets [27]. The presence of F^−^ ions modulates the growth kinetics by forming weak coordination with metal ions, thereby suppressing nanosheet aggregation and promoting uniform morphology. Finally, N-GQDs are introduced through a secondary hydrothermal treatment, leading to their uniform anchoring on the NiFe-LDH surface. Benefiting from abundant functional groups and defect sites, N-GQDs not only regulate the local electronic structure but also enhance structural stability through strong interfacial interactions.

### 3.2. Structural Compositions and Morphologies of Catalysts

Optical photographs of catalysts (Figure S1) demonstrate that NiFe-LDH grows uniformly on the surface of NF. The phase structure and surface morphology were further characterized by XRD and SEM, respectively, as shown in Figures S2–S6. Two sharp characteristic peaks at approximately 44.5° and 51.8° are detected for all samples, which can be indexed to the (111) and (200) crystal planes of metallic Ni (PDF#04-0850), respectively. Single-component Ni and Fe catalysts are identified as metal hydroxides, matching well with Fe_6_(OH)_12_(CO_3_) (PDF#46-0098) and Ni(OH)_2_ (PDF#14-0117). All bimetallic catalysts exhibit the characteristic NiFe-LDH phase, with variations only in the Ni/Fe composition ratio. The diffraction peaks of F1N2 at 11.5°, 23.3°, 34.6°, 39.1°, and 46.4° are assigned to the (003), (006), (012), (015), and (018) planes of NiFe-LDH, respectively, in agreement with the standard card Ni_5.64_Fe_2.36_(OH)_16_(CO_3_)_1.18_·7.52H_2_O (PDF#51-0463).

**Figure 2:**
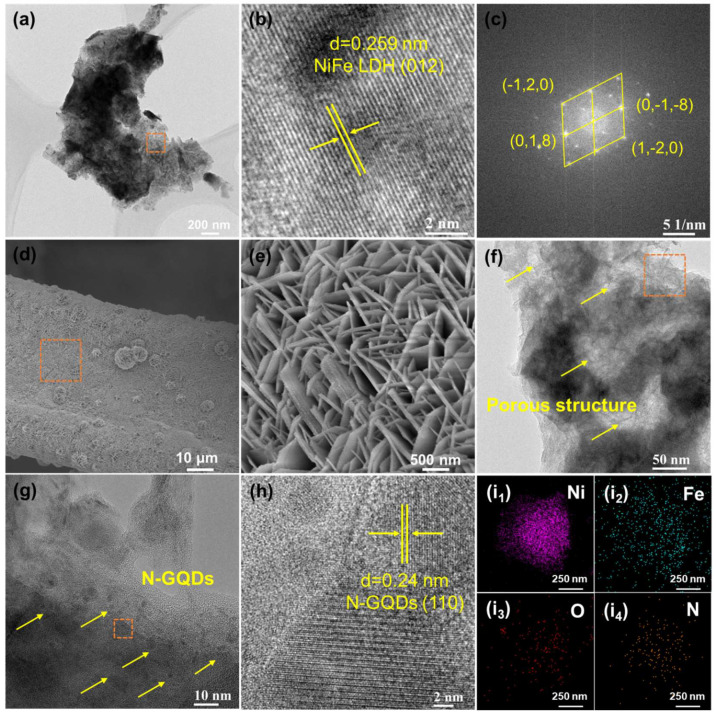
(**a**,**b**) TEM and HRTEM images of F1N2; (**c**) FFT pattern of F1N2; (**d**,**e**) SEM images of N-F1N2; (**f**–**h**) TEM and HRTEM images of N-F1N2; elemental mapping: (**i_1_**) Ni, (**i_2_**) Fe, (**i_3_**) O, (**i_4_**) N.

SEM results demonstrate that all catalysts exhibit nanosheet morphology and are uniformly distributed on NF substrates. Compared with pure Ni catalysts, Fe samples possess denser nanosheet arrays. A small quantity of nanoflower structures originates from excessive aggregation of nucleation sites, which restricts the flat spreading of nanosheets and induces self-assembly into spherical nanoflowers. For bimetallic catalysts, the size and thickness of nanosheets increase gradually with rising Fe molar ratio. When the Ni/Fe molar ratio reaches 2:1 (F1N2), uniformly distributed NiFe-LDH nanosheets are well dispersed on the NF surface. The moderate nanoflower morphology facilitates ion transport by providing a large surface area and shortened diffusion pathways. TEM was further employed to probe the crystal microstructure of NiFe-LDH. As illustrated in Figure 2a–c, F1N2 displays ultrathin wrinkled nanosheets with partial layer stacking, consistent with SEM results. HRTEM images exhibit well-preserved crystallinity of NiFe-LDH, and the lattice fringe with an interplanar spacing of 0.259 nm is assigned to the (012) lattice plane. The corresponding Fast Fourier Transform (FFT) pattern presents ordered and symmetrical diffraction spots, matching the characteristic crystal planes of NiFe-LDH.

**Figure 3:**
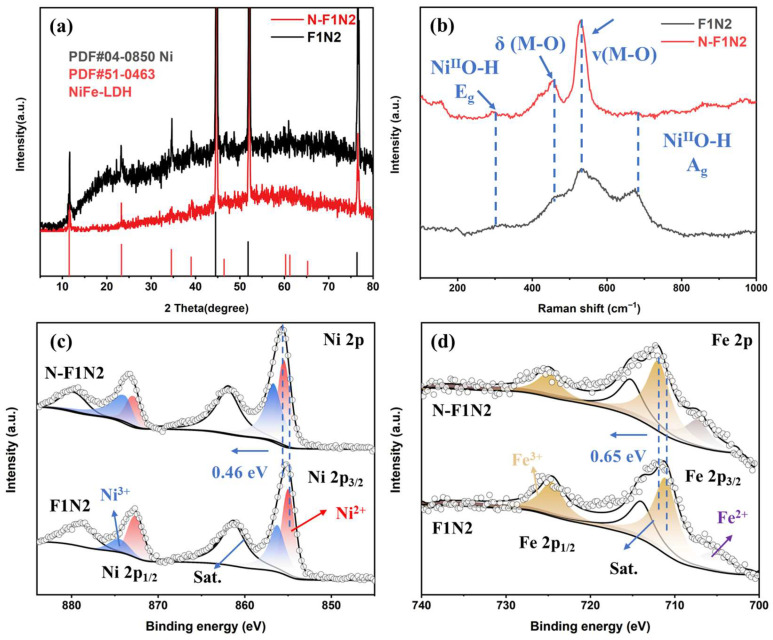
(**a**) XRD patterns, (**b**) Raman spectra of F1N2 and N-F1N2; High-resolution XPS spectra of F1N2 and N-F1N2: (**c**) Ni 2p, (**d**) Fe 2p.

N-GQDs were in situ loaded on F1N2 surfaces via a facile hydrothermal method. As shown in Figure 2d,e, N-F1N2 preserves the original morphology of F1N2, with uniformly distributed interlaced nanosheets on the NF substrate. No significant changes are observed in the XRD patterns of N-F1N2, suggesting that the hydrothermal deposition process did not affect the phase structure (Figure 3a). TEM images of N-F1N2 (Figure 2f) reveal abundant pore structures on NiFe-LDH nanosheets, which can increase specific surface area, improve electrolyte wettability and reduce interfacial impedance [28]. As shown in Figure 2g, uniformly dispersed N-GQDs on NiFe-LDH (The arrows highlight the presence of N-GQDs on the catalyst surface) remarkably enhance electrical conductivity and interfacial electron transfer kinetics. The lattice fringe with 0.24 nm interplanar spacing in HRTEM (Figure 2h) corresponds to the (110) lattice plane of N-GQDs, verifying successful incorporation of N-GQDs. Moreover, the EDS mappings demonstrate the homogeneous distribution of Ni and Fe across the nanosheets, as shown in Figure 2(i_1_–i_4_).

### 3.3. Surface Chemical Properties of Catalysts

Raman spectra were employed to investigate the surface structures of F1N2 and N-F1N2, as illustrated in Figure 3b. The characteristic peaks at 295 cm^−1^ and 675 cm^−1^ are observed for all samples, corresponding to the bending vibration and stretching vibration of Ni^II^O-H bonds, respectively [29]. Moreover, the peaks located at 456 cm^−1^ and 532 cm^−1^ are assigned to the stretching vibration and in-plane bending vibration of laminate M-O bonds, synergistically verifying the well-ordered layered coordination crystal structure of NiFe-LDH [30]. Compared with F1N2, N-F1N2 exhibits remarkably enhanced peak intensity, demonstrating that N-GQDs optimize the metal coordination environment and active hydroxyl sites.

XPS was utilized to deeply explore the surface elemental valence states and the influence of N-GQDs on interfacial electron transfer. The survey XPS spectra (Figure S7) clearly display characteristic signals of Ni, Fe, O, C and F elements. The high-resolution Ni 2p spectra are shown in Figure 3c. The peaks at 873.4 eV and 855.6 eV correspond to Ni 2p_1/2_ and Ni 2p_3/2_ orbitals, respectively, while the satellite peaks at 879.7 eV and 861.9 eV indicate the coexistence of multiple nickel valence states. Compared with F1N2, N-F1N2 exhibits a higher proportion of Ni^3+^ species, accompanied by a positive shift of 0.46 eV in the Ni 2p binding energy. This result indicates that N-GQDs modulate the localized electronic structure and valence reconstruction of Ni sites, induce electron transfer from Ni to N-GQDs, and strengthen the covalent hybridization of Ni-O bonds [31]. Accordingly, abundant high-valence active Ni^3+^ species are accumulated to accelerate OER kinetics. As shown in high-resolution Fe 2p spectra (Figure 3d), the characteristic peaks at 712.1 eV and 724.9 eV are attributed to Fe^3+^, and the peak at 707.2 eV belongs to Fe^2+^. The contents of Fe^3+^ and Fe^2+^ in N-F1N2 are calculated to be 72.32% and 27.68%, respectively. Abundant Fe^3+^ sites can provide sufficient active centers for catalytic reactions [32]. In addition, the Fe 2p binding energy of N-F1N2 shifts toward higher energy by 0.65 eV relative to F1N2, manifesting that N-GQDs reduce the electron density around Fe sites and promote the oxidation of Fe^2+^ to highly active Fe^3+^. Such electronic redistribution may contribute to enhanced interfacial charge transfer kinetics during the OER process. Combined with the reduced charge transfer resistance observed from EIS analysis and the Raman results, these findings suggest strengthened interfacial electronic coupling after the introduction of N-GQDs. The synergistic Ni-Fe electronic regulation further accelerates interfacial charge transfer dynamics.

In summary, strong interfacial electronic coupling is constructed between N-GQDs and NiFe-LDH, which differentially regulates valence evolution and localized electron distribution of bimetallic Ni and Fe sites. Electrons migrate and redistribute on Ni sites to stabilize high-valence Ni^3+^ active species, while decreased electron density on Fe sites drives efficient oxidation of low-valence Fe^2+^ into highly active Fe^3+^. Together with abundant hydroxyl active sites and an ordered layered coordination environment, the synergistic effect effectively facilitates proton-coupled electron transfer, intermediate adsorption/desorption, and lattice oxygen oxidation during OER. As a result, the alkaline OER activity and reaction kinetics are effectively improved.

### 3.4. Electrocatalytic Performance of Catalysts

**Figure 4:**
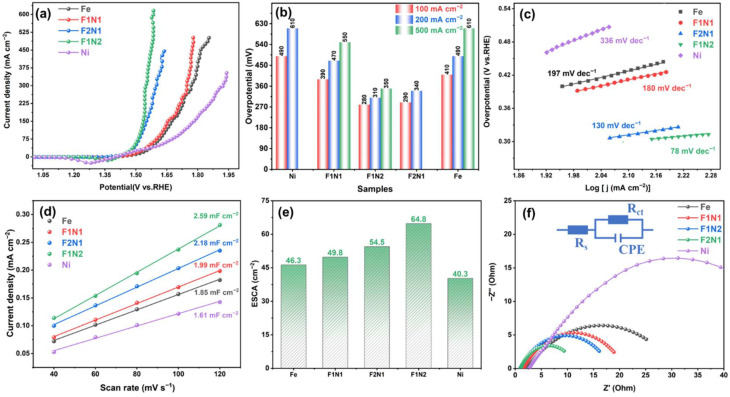
Electrochemical OER performances of catalysts: (**a**) LSV curves, (**b**) overpotential, (**c**) Tafel slopes, (**d**) Cdl, (**e**) ECSA and (**f**) EIS Nyquist curves.

The OER performances of catalysts with different Ni/Fe molar ratios were evaluated using a standard three-electrode system to determine the optimal catalyst composition. According to LSV curves of catalysts with diverse Ni/Fe ratios in 1 M KOH (Figure 4a), monometallic catalysts (Ni and Fe) deliver much worse performance than bimetallic counterparts. Among monometallic samples, the Fe-based catalyst shows higher OER activity than the Ni-based catalyst, which is mainly attributed to the higher intrinsic OER activity of Fe sites and easier formation of highly active high-valence species [33]. Compared with other bimetallic catalysts, F1N2 exhibits the lowest initial potential and the highest current density at identical applied potentials. The overpotentials of F1N2 are 280, 310 and 350 mV at current densities of 100, 200 and 500 mA cm^−2^, respectively, which are distinctly lower than those in other control samples (Figure 4b). These results indicate that the bimetallic synergistic effect significantly reduces OER reaction energy barriers, maintains ultralow OER overpotential under large-current conditions and possesses the optimal catalytic reaction kinetics. As shown in Figure 4c, F1N2 possesses the smallest Tafel slope, demonstrating the fastest interfacial charge transfer rate. The Tafel slopes of Fe, F1N1, F2N1 and Ni catalysts are 197, 180, 130 and 336 mV dec^−1^, respectively.

To quantitatively calculate the number of effective OER active sites on catalyst surfaces and reveal intrinsic catalytic activity, CV measurements were performed at scan rates of 40–120 mV s^−1^ within the non-Faradaic region (Figure S8). The Cdl values were derived from the linear fitting of the current density difference versus scan rate (Figure 4d). The Ni and Fe catalysts exhibit relatively low Cdl values of 1.61 and 1.85 mF cm^−2^, respectively, whereas the bimetallic catalysts show higher values, consistent with the LSV results. Specifically, the Cdl values of F1N1, F2N1, and F1N2 are 1.99, 2.18, and 2.59 mF cm^−2^, respectively. The ECSA was further calculated according to Equation (2), as shown in Figure 4e. F1N2 possesses the largest ECSA of 54.8 cm^2^, which fully exposes OER active sites, shortens ion transport paths and reduces interfacial impedance, thereby accelerating electrochemical reactions. The ECSA values of Fe, F1N1, F1N2 and Ni catalysts are 46.3, 49.8, 54.5 and 40.3 cm^2^, respectively.

EIS was further employed to analyze interfacial impedance and electron transfer kinetics (Figure 4f). All Nyquist plots present a single semicircle dominated by charge transfer in the high-frequency region [34]. The X-axis intercept corresponds to the equivalent series resistance (R_s_), whereas the semicircle diameter reflects the charge transfer resistance (R_ct_). Ni catalyst displays the largest interfacial resistance, with R_s_ and R_ct_ being 2.77 Ω and 53.16 Ω, respectively, which severely hinders electron transfer efficiency (Table S1).

As the Fe molar ratio increases, R_s_ and R_ct_ continuously decrease. The R_s_ values of Fe, F1N1 and F1N2 catalysts are 2.26, 1.58 and 1.03 Ω, respectively. Notably, the R_s_ and R_ct_ of F1N2 are 0.98 and 9.45 Ω, respectively, indicating that the electrolyte ion conductivity efficiency is higher, which can improve the electron transfer efficiency and accelerate the OER catalytic reaction kinetics. In summary, F1N2 with an optimal Ni/Fe atomic ratio relies on strong Ni-O-Fe interfacial electronic coupling synergy. It integrates ultralow OER overpotential, small Tafel slope, large ECSA and low interfacial impedance, efficiently accumulates high-valence Ni^3+^/Fe^3+^ active species, and synchronously accelerates proton migration, interfacial electron transport and intermediate conversion processes, showing competitive OER performance under alkaline large-current operating conditions.

### 3.5. Electrocatalytic Performance of N-F1N2

**Figure 5:**
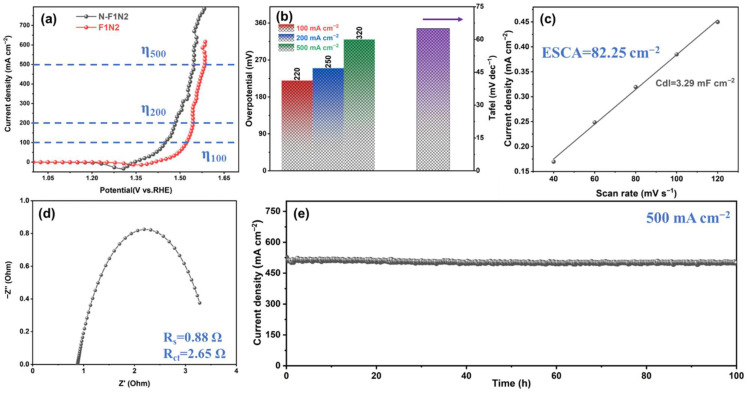
Electrochemical OER characterization of N-F1N2, including (**a**) LSV curves, (**b**) overpotentials and Tafel slopes, (**c**) Cdl and ECSA values, (**d**) Nyquist plots, and (**e**) chronoamperometric stability at a constant potential of 1.55 V vs. RHE in 1 M KOH at 25 °C without iR compensation.

N-GQDs were further incorporated onto F1N2 through in situ hydrothermal treatment to enhance the OER activity and stability, and the resulting electrocatalytic performance was systematically evaluated. LSV curves of N-F1N2 and F1N2 are presented in Figure 5a. Compared with pristine F1N2, N-F1N2 shows a negatively shifted OER onset potential and a substantially higher current density under the same applied potential. The overpotentials at 100, 200, and 500 mA cm^−2^ are as low as 220, 250, and 320 mV, respectively, remarkably outperforming those for F1N2. In addition, N-F1N2 exhibits an ultralow Tafel slope of 65 mV dec^−1^, indicating accelerated reaction kinetics. This result demonstrates that the interfacial coupling of N-GQDs modulates the localized electronic structure of NiFe-LDH, reduces the OER reaction energy barriers and accelerates OER reaction kinetics in alkaline media.

The incorporation of N-GQDs significantly enlarges the ECSA of N-F1N2 to 82.25 cm^2^, indicating increased exposure of electrochemically active sites (Figure 5c and Figure S9). The reduced R_s_ and R_ct_ values indicate accelerated charge transfer kinetics in N-F1N2, arising from the conductive N-GQD network that facilitates interfacial electron transport (Figure 5d). Chronoamperometric tests were performed at a high current density of 500 mA cm^−2^ to evaluate the practical applicability of N-F1N2 (Figure 5e). Notably, N-F1N2 maintains stable operation for 100 h with minimal current attenuation, retaining 98% of the initial current density. This indicates that N-GQDs can stabilize the crystal structure of NiFe-LDH, suppress the loss of active sites, and endow the catalyst with excellent long-term durability under large-current conditions, thus showing potential for industrial application. To further evaluate the practical significance of the developed catalyst, the OER performance of N-F1N2 was compared with representative recently reported electrocatalysts and commercial noble-metal catalysts, as summarized in Table S2. The comparison demonstrates that N-F1N2 exhibits competitive activity.

**Figure 6:**
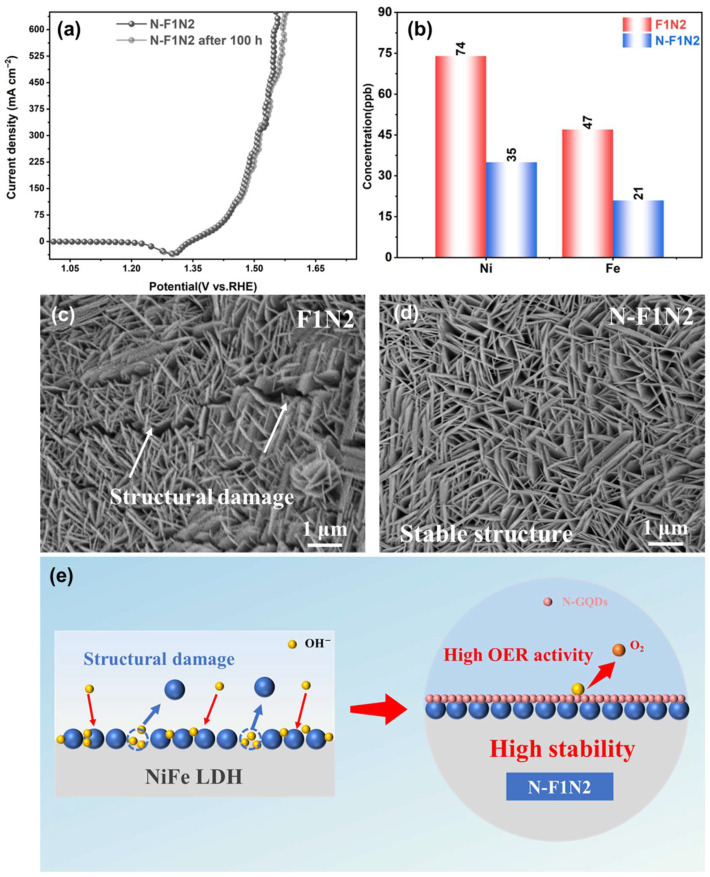
(**a**) LSV curves of N-F1N2 before and after 100 h test, (**b**) metal ion content in the electrolyte of N-F1N2 and F1N2; SEM images of after stability test: (**c**) F1N2, (**d**) N-F1N2; (**e**) mechanism diagram of N-GQDs enhancing OER stability of N-F1N2.

The LSV curves before and after the 100 h durability test exhibit negligible changes, confirming the excellent electrochemical stability of N-F1N2 (Figure 6a). The LSV curves are highly overlapped with no obvious degradation in catalytic performance, fully confirming that N-F1N2 possesses both excellent catalytic activity and long-term stability. ICP-MS measurements (Figure 6b) were performed to quantitatively analyze the metal ion leaching in the electrolyte after the stability test. Compared with F1N2, the leaching amounts of Ni and Fe ions in the electrolyte of N-F1N2 are significantly reduced, reaching only 35 ppb and 21 ppb, respectively. To further verify the structural stability of N-F1N2 during long-term operation, XRD characterization was performed after the 100 h stability test. As shown in Figure S10, no obvious changes in the diffraction peaks are observed, indicating that the layered structure of NiFe-LDH is well preserved. This result further confirms that the introduction of N-GQDs contributes to stabilizing the crystal structure during prolonged electrolysis. This indicates that N-GQDs can effectively anchor bimetallic active sites, suppress metal ion leaching, and ensure the long-term structural stability of the catalyst. Figure 6c,d show the SEM images of F1N2 and N-F1N2 after the stability test, respectively. F1N2 exhibits obvious irreversible structural damage after the test, such as significant nanosheet aggregation, structural rupture and collapse. In contrast, N-F1N2 still maintains an intact structure with no significant morphological change.

In summary, N-F1N2 exhibits the most excellent OER catalytic activity and stability. Combined with the characterization results of SEM, TEM and XPS, its outstanding performance (Figure 6e) can be attributed to the following aspects: (1) The uniformly dispersed NiFe-LDH nanosheets on N-F1N2 can enhance electrolyte wettability and improve ion diffusion rate. Meanwhile, the well-crystallized structure of N-F1N2 provides ideal channels for electron transport, substantially improving charge transfer efficiency. (2) Interfacial electronic coupling between N-GQDs and Ni/Fe sites modulates the local electronic structure and valence states, thereby enhancing charge transfer and accelerating OER kinetics. (3) N-GQDs further stabilize the layered NiFe-LDH architecture by suppressing structural degradation and metal dissolution under prolonged high-current operation, leading to superior electrochemical durability. (4) The abundant surface functional groups and defect sites of N-GQDs provide abundant active sites for OER while simultaneously reducing interfacial resistance and facilitating mass transport.

## 4. Conclusions

In summary, a highly efficient N-GQDs@NiFe-LDH composite electrocatalyst was successfully constructed on NF through a facile two-step hydrothermal strategy. By optimizing the Ni/Fe molar ratio, F1N2 exhibits optimal intrinsic OER activity among all samples. The incorporation of N-GQDs further enhances the electrocatalytic activity and structural stability of NiFe-LDH through strong interfacial electronic coupling and confinement effects. As a result, the optimized N-F1N2 catalyst achieves low overpotentials of 220, 250, and 320 mV at current densities of 100, 200, and 500 mA cm^−2^, respectively, together with a low Tafel slope of 65 mV dec^−1^. More importantly, N-F1N2 demonstrates improved long-term durability, retaining 98% of its initial current density after continuous operation at 500 mA cm^−2^ for 100 h. Comprehensive characterizations reveal that N-GQDs effectively modulate the valence states of Ni/Fe active sites, enrich highly active Ni^3+^/Fe^3+^ species, accelerate interfacial charge transfer, enlarge the electrochemically active surface area, and stabilize the layered NiFe-LDH structure against metal dissolution and structural collapse. The developed N-GQD-modified NiFe-LDH catalyst exhibits strong potential for practical industrial water electrolysis, providing valuable insights into the design of advanced non-noble-metal OER electrocatalysts.

## Data Availability

The original contributions presented in this study are included in the article and Supplementary Materials. Further inquiries can be directed to the corresponding author.

## Supplementary Materials

The following supporting information can be downloaded at https://www.mdpi.com/article/10.3390/ma19102081/s1: Figure S1: Digital optical photos of the synthesized catalysts; Figure S2: (a) XRD pattern, (b,c) SEM images of Fe-based catalysts; Figure S3: (a) XRD pattern, (b,c) SEM images of F1N1; Figure S4: (a) XRD pattern, (b,c) SEM images of F2N1; Figure S5: (a) XRD pattern, (b,c) SEM images of F1N2-based catalysts; Figure S6: (a) XRD pattern, (b,c) SEM images of Ni-based catalysts; Figure S7: Full XPS spectra of F1N2 and N-F1N2; Figure S8: CV curves of catalysts; Figure S9: CV curves of N-F1N2; Figure S10: The XRD pattern of N-F1N2 after 100 h stability test. Table S1. The R_s_ and R_ct_ of catalysts; Table S2. Performance comparison of representative OER electrocatalysts.
