# Supplementary material for: Highly Dispersed N-Doped Graphene Quantum Dot-Assisted NiFe Bimetallic Sites for Efficient Water Oxidation
Source: Materials (Basel). 2026 May 15;19(10):2081. doi: 10.3390/ma19102081 (PMC13208120; doi:10.3390/ma19102081)
Supplement: Supplementary file 1 [file materials-19-02081-s001.zip › materials-4311461-supplementary.pdf]

Highly Dispersed N-Doped Graphene Quantum Dots Assisted NiFe Bimetallic Sites for Efficient Water Oxidation

Yongbo Wang [1,†], Xin Jin[1,†], Yanfei Fan[1], Guanwei Cui [1,*] and Bo Tang[1,2]

1 College of Chemistry, Chemical Engineering and Materials Science, Shandong Normal University, Jinan 250014, China; wyongbo1011@163.com

2 Laoshan Laboratory, Qingdao, Shandong, China; tangbo0765@163.com

* Correspondence: Cguanwei0812@163.com

† These authors contributed equally to this work

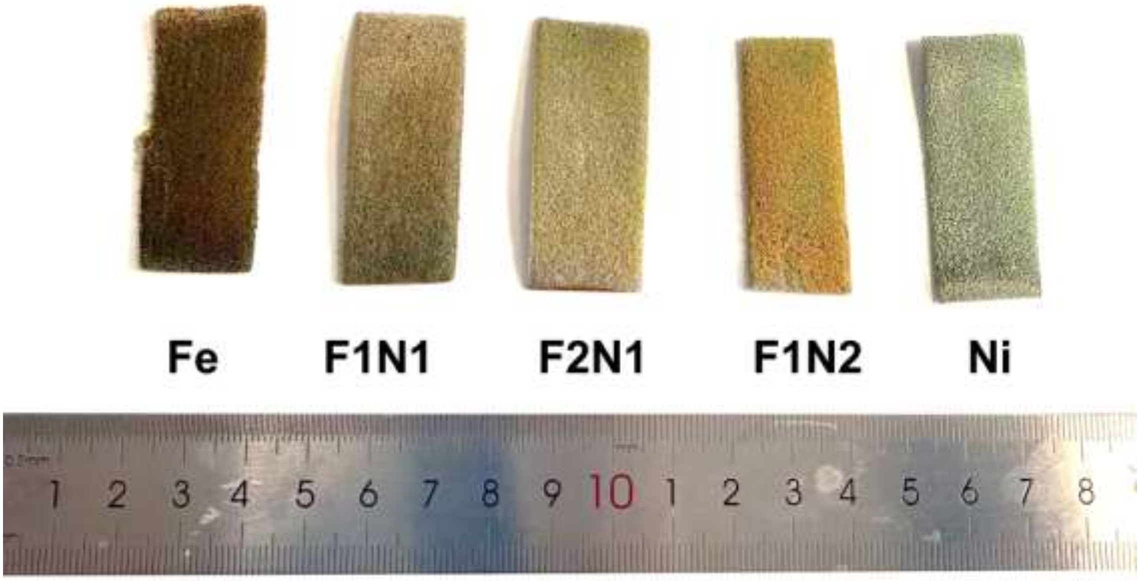

Figure S1 Digital optical photos of the synthesized catalysts

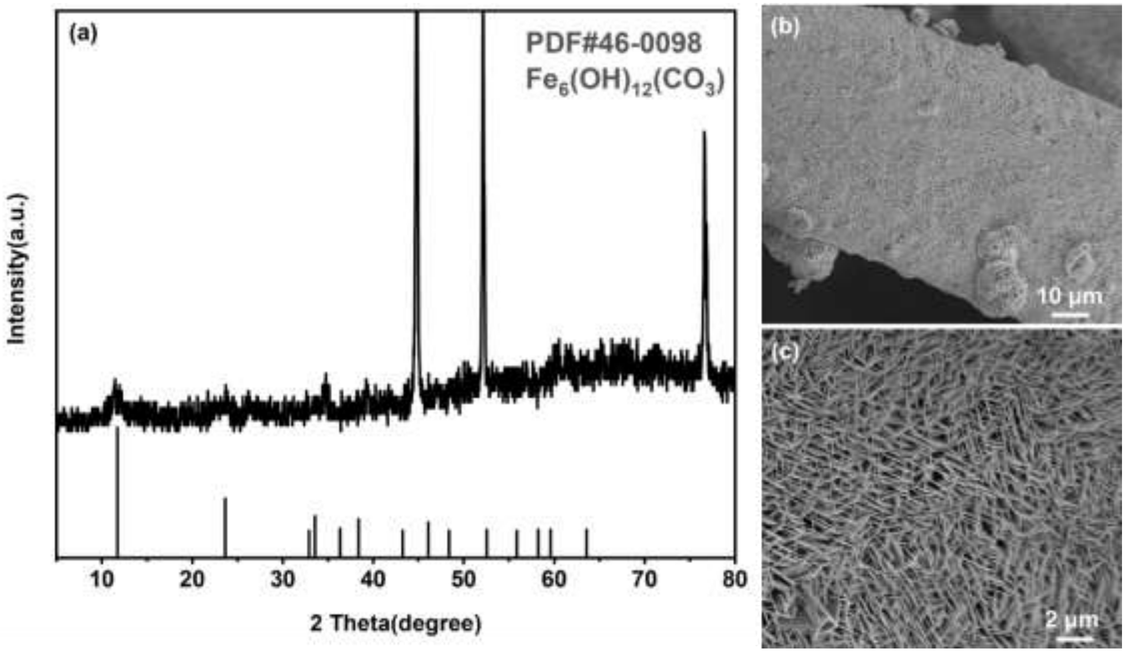

Figure S2 (a) XRD pattern, (b-c) SEM images of Fe based catalysts

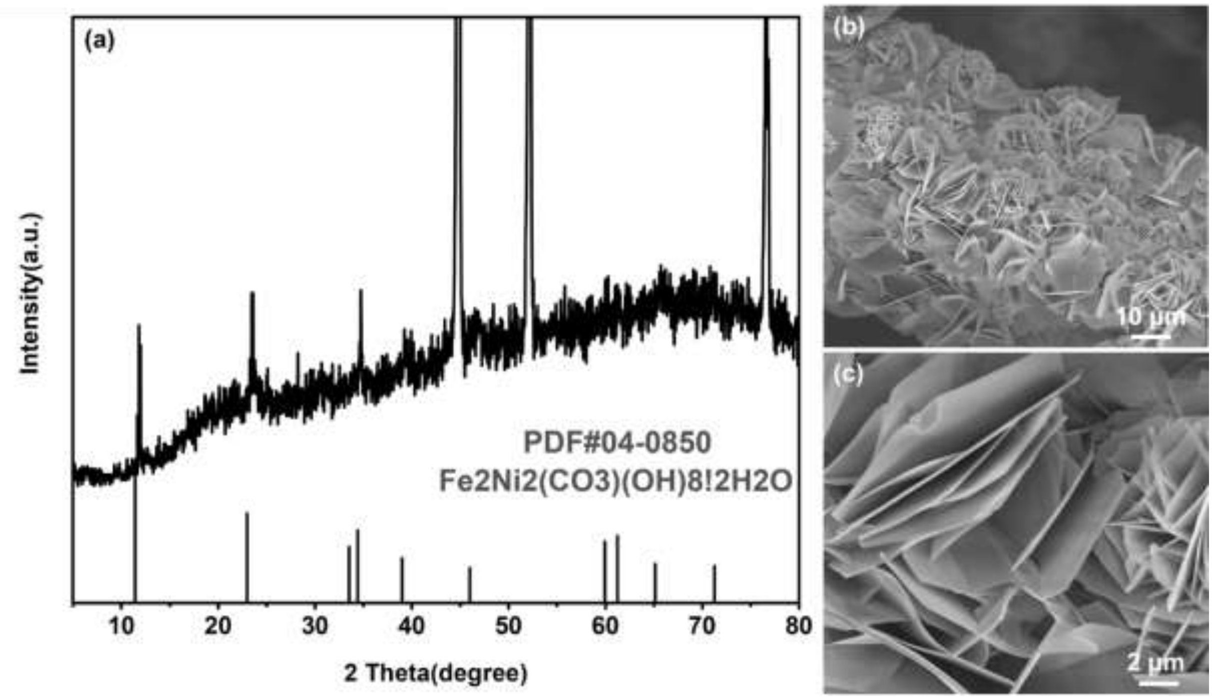

Figure S3 (a) XRD pattern, (b-c) SEM images of F1N1

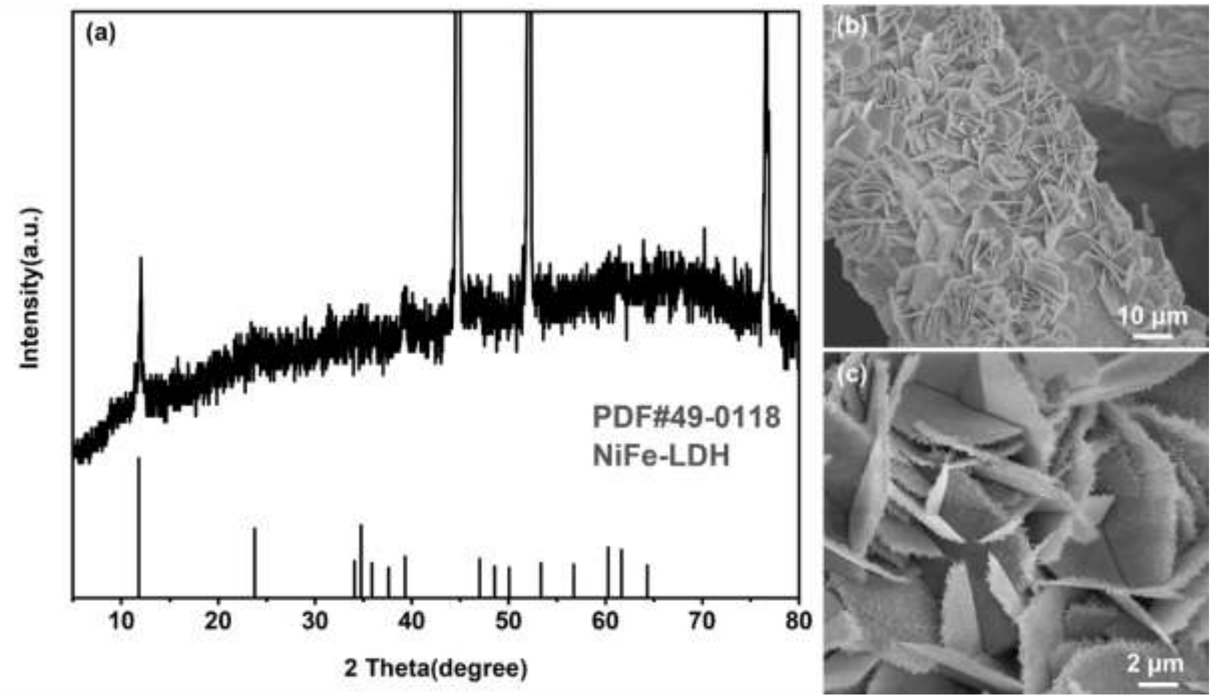

Figure S4 (a) XRD pattern, (b-c) SEM images of F2N1

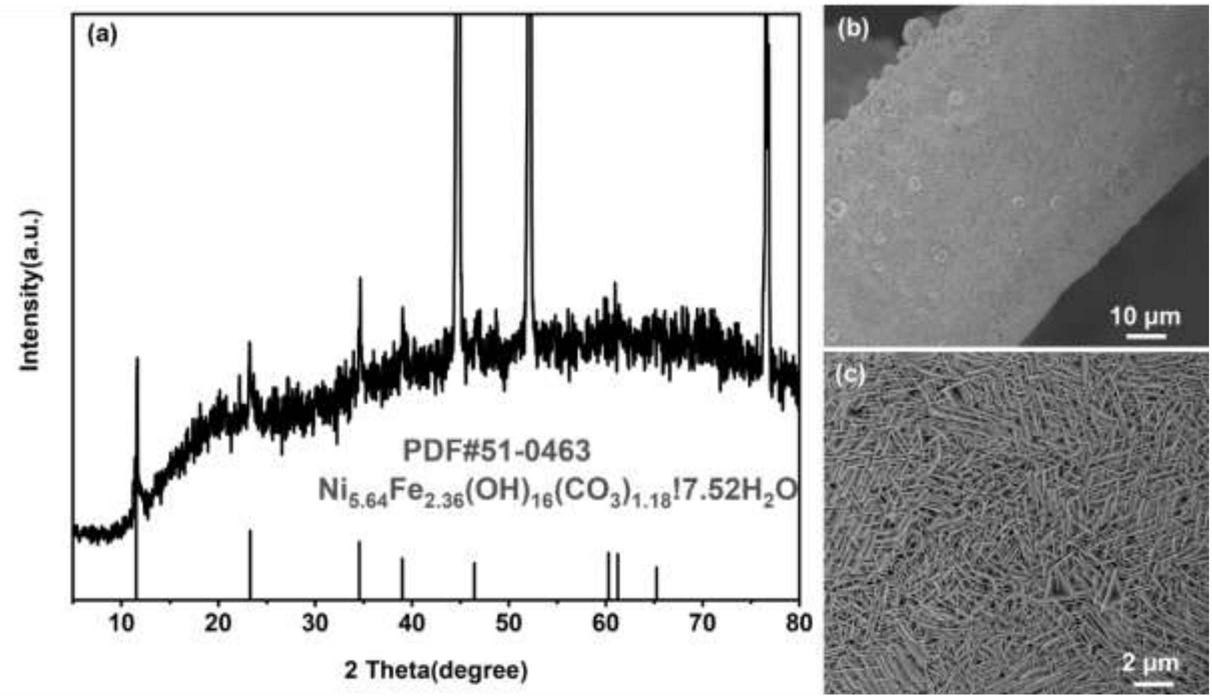

Figure S5 (a) XRD pattern, (b-c) SEM images of F1N2 based catalysts

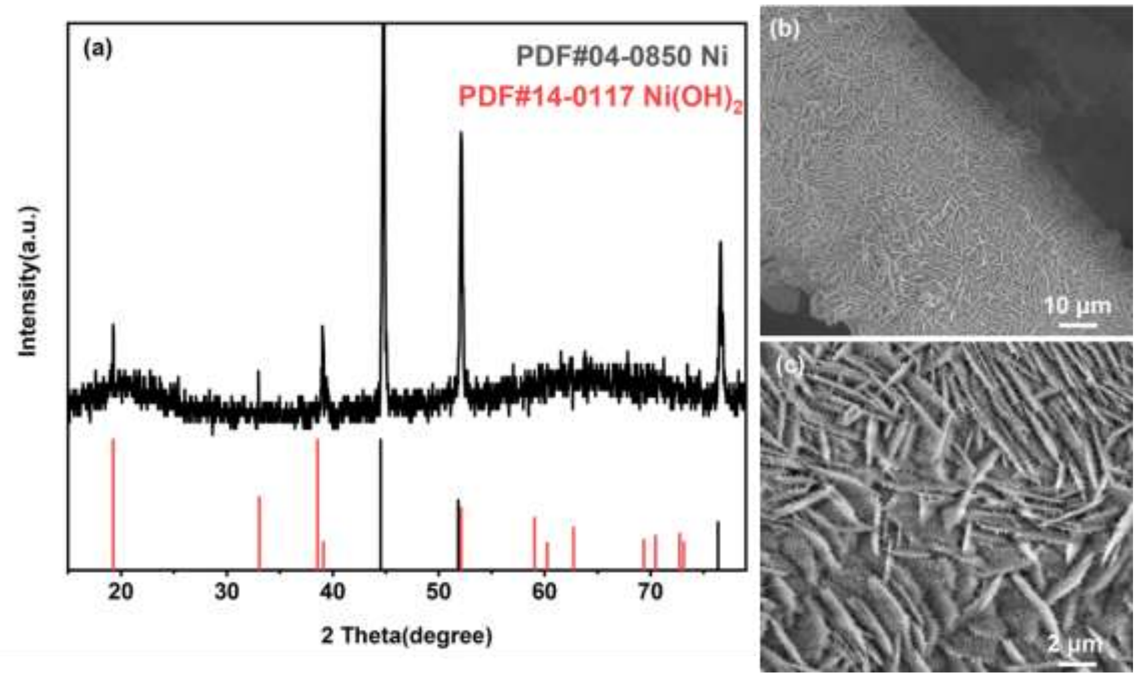

Figure S6 (a) XRD pattern, (b-c) SEM images of Ni based catalysts

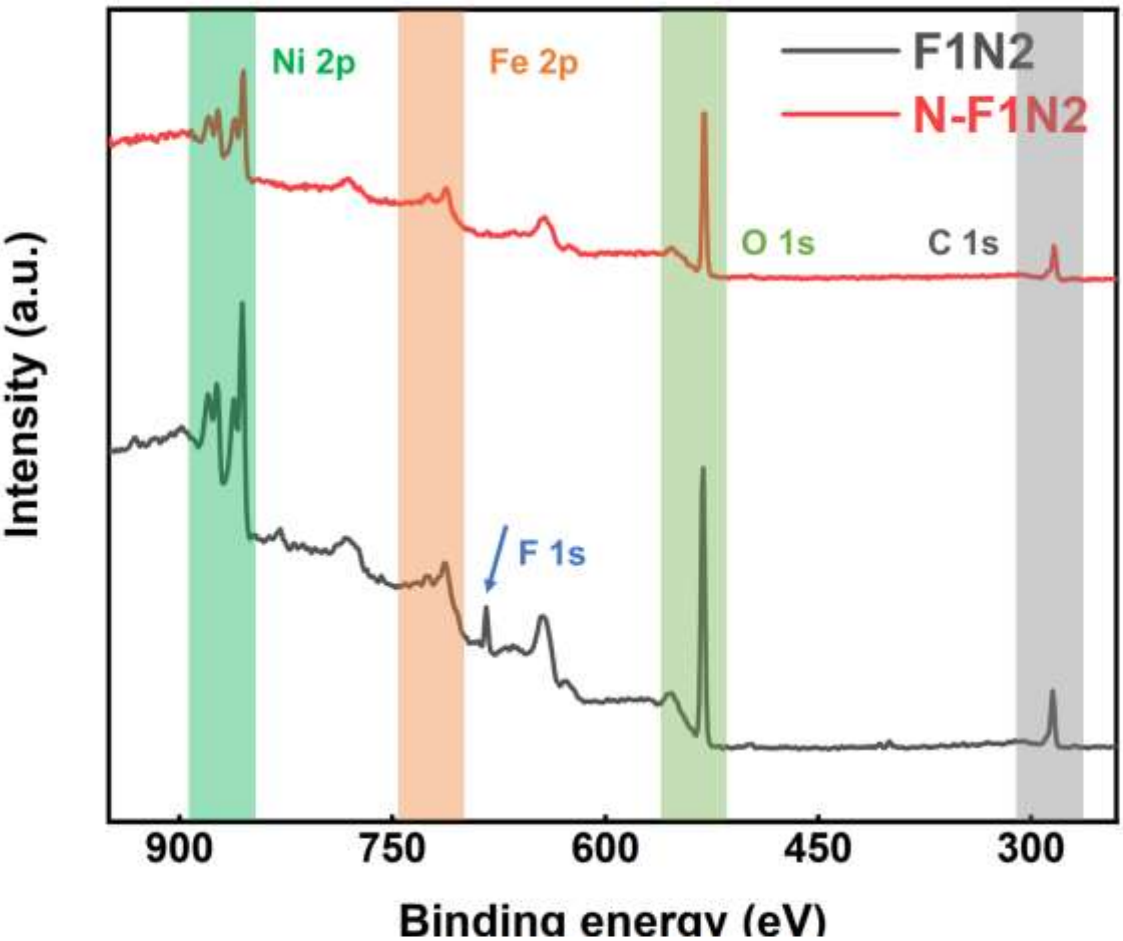

Figure S7 Full XPS spectra of F1N2 and N-F1N2

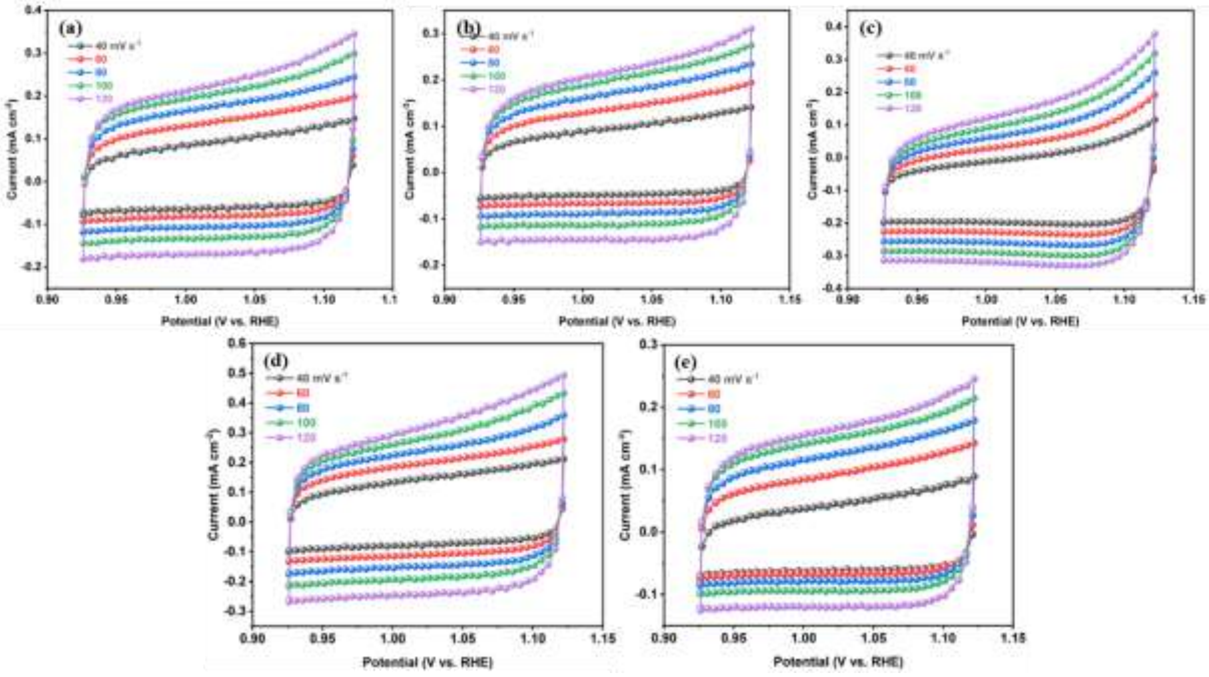

Figure S8 CV curves of catalysts

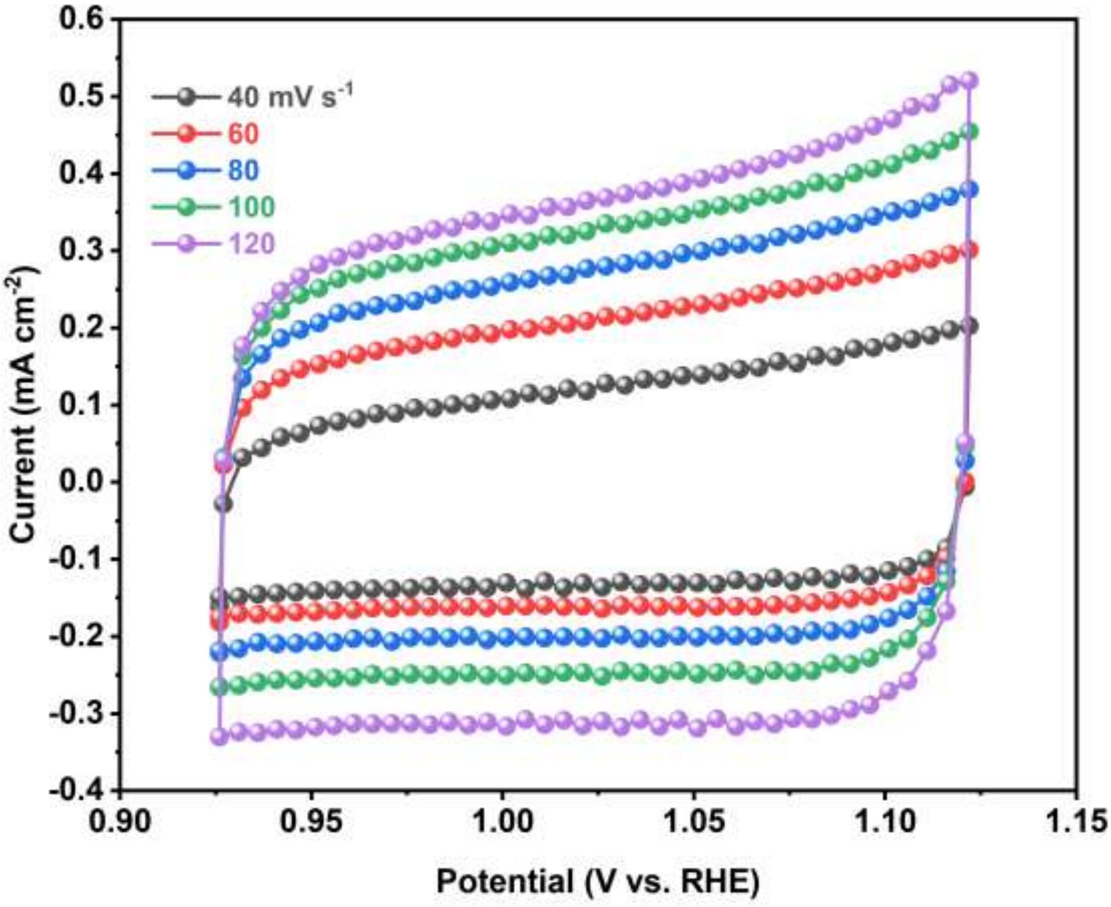

Figure S9 CV curves of N-F1N2

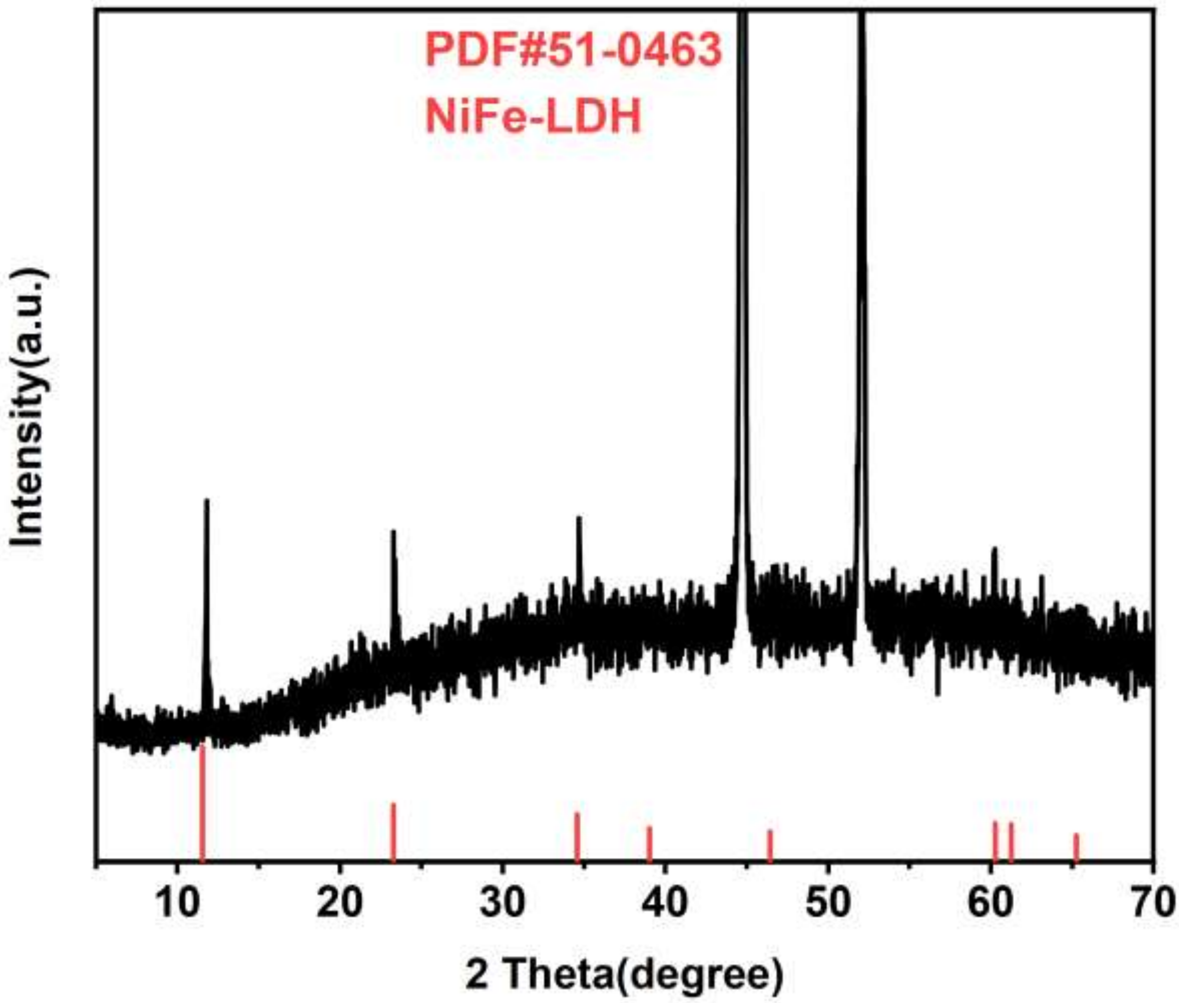

Figure S10 the XRD pattern of N-F1N2 after 100 h stability test

Table S1 The Rs and Rct of catalysts

Samples	R_s (Ω)	R_{ct} (Ω)
Fe	2.26	28.17
F1N1	1.58	20.02
F1N2	1.03	14.68
F2N1	0.98	9.45
Ni	2.77	53.16

Table S2 Performance comparison of representative OER electrocatalysts

**Catalyst**	**Electrolyte**	**η@100 mA cm^{-2}**	**Ref**
N-F1N2	1 M KOH	220	This work
Ru-Ni(Fe)P2/NF	1 M KOH	251	Small. 19, 2300030 (2023)
Ru-CoOx/NF	1 M KOH	220	Small. 17, 2102777 (2021)
Ta-NiFe LDH	1 M KOH	283	Chem Eng J. 403, 126297 (2021)
NiFeCr LDH/NF	1 M KOH	279	Appl Catal B: Environ Energy. 383, 126109 (2026)
NiSe@NiMn LDH/NF	1 M KOH	287	J Colloid Interface Sci. 658, 528-539 (2024).
Ag/NiFe LDH	1 M KOH	269	Nano Energy. 98, 107212 (2022)
Ce-NiFe LDH	1 M KOH	250	J. Energy Chem. 91, 306-312 (2024)
RuO2	1 M KOH	390	Nat Commun 16, 8788 (2025)
